# PhenoMeter: A Metabolome Database Search Tool Using Statistical Similarity Matching of Metabolic Phenotypes for High-Confidence Detection of Functional Links

**DOI:** 10.3389/fbioe.2015.00106

**Published:** 2015-07-29

**Authors:** Adam J. Carroll, Peng Zhang, Lynne Whitehead, Sarah Kaines, Guillaume Tcherkez, Murray R. Badger

**Affiliations:** ^1^College of Medicine, Biology and Environment, Research School of Biology, The Australian National University, Canberra, ACT, Australia

**Keywords:** metabolomics, metabolic phenotyping, pattern recognition, metabolomics database, search algorithm, phenomics

## Abstract

This article describes PhenoMeter (PM), a new type of metabolomics database search that accepts metabolite response patterns as queries and searches the MetaPhen database of reference patterns for responses that are statistically significantly similar or inverse for the purposes of detecting functional links. To identify a similarity measure that would detect functional links as reliably as possible, we compared the performance of four statistics in correctly top-matching metabolic phenotypes of *Arabidopsis thaliana* metabolism mutants affected in different steps of the photorespiration metabolic pathway to reference phenotypes of mutants affected in the same enzymes by independent mutations. The best performing statistic, the PM score, was a function of both Pearson correlation and Fisher’s Exact Test of directional overlap. This statistic outperformed Pearson correlation, biweight midcorrelation and Fisher’s Exact Test used alone. To demonstrate general applicability, we show that the PM reliably retrieved the most closely functionally linked response in the database when queried with responses to a wide variety of environmental and genetic perturbations. Attempts to match metabolic phenotypes between independent studies were met with varying success and possible reasons for this are discussed. Overall, our results suggest that integration of pattern-based search tools into metabolomics databases will aid functional annotation of newly recorded metabolic phenotypes analogously to the way sequence similarity search algorithms have aided the functional annotation of genes and proteins. PM is freely available at MetabolomeExpress (https://www.metabolome-express.org/phenometer.php).

## Introduction

The emergence of high throughput omics technologies has driven an explosive increase in the global rate of biological data generation. Recognizing the urgent need to systematically capture and preserve these data for future re-use, the scientific community has been responding to the data explosion by developing minimal reporting standards, standardized data sharing formats, and databases for a wide variety of technologies and research fields (Fiehn et al., 2008; Taylor et al., 2008; Field et al., 2010; Fernie et al., 2011). As the challenges of capture, storage, and exchange are overcome in new fields, we anticipate increased efforts to make accumulated data more immediately useful to biologists by equipping databases with advanced analytical capabilities extending beyond simple search, browse, and visualization functions. In this article, we describe one such effort in the field of metabolomics to turn a database of experimentally observed metabolite responses into an analytical tool by equipping it with metabolite response pattern-based search functionality.

A well-known example of an added-value metabolomics database is the Golm Metabolome Database (GMD) (Kopka et al., 2005). The earliest GMD versions focused on sharing reference mass-spectral and retention index (MSRI) reference libraries for gas chromatography/mass spectrometry (GC/MS) peak identification, providing basic browse and text search options. Subsequent versions added value to these libraries by introducing new tools for MSRI data-based search with chemical sub-structure prediction so users can search their own spectra against the collection of reference spectra to identify their peaks and, in the case of unmatched spectra, gain clues about possible chemical structures (Hummel et al., 2010).

The GMD developers recently introduced new features to support the storage and visualization of experimental metabolite level data, the comparison of metabolite levels between different experimental treatments and a novel “profile search” tool that searches experimental metabolite level profiles (signal intensities of metabolites within a single sample class) against a database of reference profiles to find similar profiles using a dot-product similarity measure.^1^ To our knowledge, formal descriptions of these latest features are not yet published. Nevertheless, by developing the Profile Search feature, the GMD has become one of the first metabolomics databases to provide *biological* pattern-based search capabilities and thereby evolve beyond being simply a well-indexed archive of biological information with basic search, browse, and visualization functions to become a biological analytical/annotation tool in the truest sense, analogous to a nucleotide sequence database equipped with a nucleotide sequence similarity search algorithm.

In the fields of transcriptomics and proteomics, a form of pattern-based database searching known as enrichment analysis (EA) has become extremely popular. The widespread uptake of EA to aid interpretation of gene product expression profiles is strongly evident in the literature. In their review, Huang et al. (2009) identified some 68 different software tools for performing EA on transcriptomic/proteomic data (Huang et al., 2009). At the time of publication, the article describing the use of DAVID, one of the earliest EA tools based on Fisher’s Exact Test, had been cited over 3000 times (Huang et al., 2008). The article describing the first example of Gene Set Enrichment Analysis (GSEA; using a Kolmogorov–Smirnov test) had been cited over 6000 times (Subramanian et al., 2005). Other examples of database-driven tools and approaches matching signature patterns within gene expression profiles for the purpose of inferring functional links include The Connectivity Map (Lamb et al., 2006), OncoMine (Rhodes et al., 2004, 2007), EXALT (Wu et al., 2009), SIGNATURE (Chang et al., 2011), ExpTreeDB (Ni et al., 2014), LINCS Canvas Browser (Duan et al., 2014), Galahad (Laenen et al., 2015), AtCAST (Kakei and Shimada, 2015), Drug-Path (Zeng et al., 2015), and NFFinder (Setoain et al., 2015). Clearly, there is very active development and strong uptake within the omics community of tools that can help make sense of molecular expression profiles by matching query patterns to large databases of reference patterns.

In contrast to the field of transcriptomics, there are currently few examples of EA and no published tool allowing a public repository of metabolite response patterns to be searched on the basis of a query response pattern. Several properties of metabolites promise to make the development of metabolite response pattern-based database search tools particularly rewarding:

1. Metabolite contents provide an integrated readout of processes occurring at the transcriptional, translational, and physicochemical levels of biochemical organization and are therefore of great diagnostic utility.
2. Acquisition of untargeted metabolomics data is generally relatively inexpensive and high-throughput compared to acquisition of proteomic and transcriptomic data.
3. Many metabolites are common to different organisms and measuring them in new model systems does not require costly genome or transcriptome sequencing.

However, metabolites also present unique challenges for the development of pattern-based database search tools:

1. Relative to gene identifiers, metabolite identifiers are poorly standardized across the literature – a single metabolite is often referred to by many different identifiers in the literature thus making mapping more difficult for developers.
2. Metabolomics analyses often detect many metabolites that, despite being of biological importance and diagnostic value, are structurally unidentified.
3. Comparably few public metabolomics databases are designed to store experimentally observed metabolic phenotypes.
4. Submission of metabolomics datasets to public databases is not enforced by journals to the same extent as submission of transcriptomics and proteomics data.
5. As a consequence of points 3 and 4, systematically annotated reference metabolic phenotypes are less readily available than transcriptomic or proteomic datasets.

Despite the above challenges, examples of EA tools for metabolomics that examine query metabolite profiles for evidence of significantly coordinated responses of biologically meaningful metabolite sets stored in a database have begun to appear in recent years. For example, Xia and Wishart (2010, 2011a) have developed a web-application named metabolite set enrichment analysis (MSEA) which uses the “globaltest” algorithm, originally designed for transcriptomic EA (Goeman et al., 2004), to provide two types of EA for metabolomics:

1. Over-representation analysis (ORA, Class I EA) that checks whether any predefined metabolite sets (e.g., metabolites from certain metabolic pathways, from certain locations or with certain disease associations) are enriched in a query metabolite set.
2. Quantitative Enrichment Analysis (QEA, Class II EA) in which metabolite concentration profiles for a number of subjects differing in some continuous or discrete experimental factor of interest (e.g., drug dose) are provided as input and the “globaltest” algorithm is used to test whether any of the predefined metabolite sets are significantly differentially expressed as a group.

More recently, Xia and Wishart integrated their “globaltest” based MSEA implementation into their powerful MetaboAnalyst metabolomics data analysis web application (Xia and Wishart, 2011b) while Persicke et al. (2012) integrated an MSEA feature based on the original GSEA statistical approach (Subramanian et al., 2005) into their versatile MeltDB metabolomics data processing pipeline (Persicke et al., 2012).

While statistical methods are obviously important, the results of any EA tool can only be as meaningful as the transcript/protein/metabolite sets defined in its database. It is therefore unfortunate that creation of meaningful sets is so labor intensive. The derivation of metabolite sets from online pathway databases, while amenable to scripting, still requires manual curation, especially when the pathway database itself has not been extensively curated or was not made specifically for the organism being studied (Persicke et al., 2012). Even more laborious is the creation of metabolite sets from information scattered across the literature in a multitude of formats (Xia and Wishart, 2010). This may explain why most EA tools only provide built-in reference sets for one taxonomic group or interest area.

In this article, we describe the design and validation of MetabolomeExpress PhenoMeter – a new tool for detecting biologically meaningful patterns within metabolite response profiles. The PhenoMeter (PM) differs from metabolomics EA tools in the following ways:

1. It is not concerned with enrichments of metabolite sets but with statistical similarities between experimentally observed metabolic phenotypes.
2. Rather than using a dedicated database as its source of reference data, it utilizes the MetabolomeExpress Metabolic Phenotype Database, MetaPhen (Carroll et al., 2010) – an established organism– and technology-neutral public metabolomics data repository providing persistent public storage of experimental metabolic phenotypes – thereby distributing the workload of producing reference data across the metabolomics community.
3. For both query input and reference phenotypes, it requires only the fold changes of metabolite levels under a treatment relative to a control rather than raw measurements. This makes it possible to analyze metabolite data from the many publications that do not provide raw measurements while making the setup of PM queries simpler.

Additional features include:

1. Metabolite label permutation-based estimation of hit significance.
2. Interactive annotated scatterplots of phenotype–phenotype comparisons.
3. Network graph visualization of phenotype–phenotype similarity networks.
4. Standardized and documented database import text-file format for public submissions of experimental reference datasets.

We begin by comparing the performance of four different phenotypic similarity scoring methods in correctly matching the metabolic phenotypes of *Arabidopsis thaliana* metabolism mutants to reference phenotypes of functionally equivalent mutants affected in the same enzymes by independent mutations. To test the capacity of the different methods to correctly discriminate between similar phenotypes, we used mutants affected in various steps of a metabolic pathway rather than a set of functionally less related mutants. This pathway was photorespiration – a complex, high flux pathway that detoxifies and recycles 2-phosphoglycolate, a toxic metabolite produced by Rubisco (Ribulose-1,5-bisphosphate Carboxylase/Oxygenase) – the enzyme responsible for fixing CO_2_ during ­photosynthesis – when it fixes O_2_ instead of CO_2_ through a competing reaction (Bauwe et al., 2010, 2012; Maurino and Peterhansel, 2010).

After demonstrating the high performance of the adopted scoring method, we describe the permutation-based approach used for significance testing and show that results of typical queries are returned in practical timeframes. We then demonstrate features for visualizing phenotypic similarity networks and show that metabolic phenotypes of photorespiration mutants previously published by independent groups could be matched to the relevant reference phenotypes from this study despite differences in growth conditions and analytical technologies. Finally, we present examples of matching between metabolic responses to a variety of different perturbations besides photorespiratory gene mutation using datasets from the publications of various groups. The PM and tools like it promise to enhance the objective interpretation of metabolic phenotypes by the metabolomics community. The PM and associated documentation is publicly available without registration at the MetabolomeExpress (Carroll et al., 2010) website.^2^

## Results

### Development of the phenometer phenotypic similarity scoring algorithm

The PM accepts metabolic phenotypes (sets of metabolite fold changes in a treatment relative to a control) as queries and searches a database for phenotypes that are statistically significantly similar (or inverse). The metabolic phenotype database, MetaPhen, is part of MetabolomeExpress public metabolomics database (Carroll et al., 2010). At the time of publication, MetaPhen contained 429 publicly available phenotypes from 50 peer-reviewed publications spanning 17 organisms and 15 analytical technologies. Thus, despite being biased toward the plant kingdom, the MetaPhen database is currently comparable in size and scope to other major metabolomics databases such as the United States Government National Institute of Health (NIH) Metabolomics Workbench^3^ and the European Bioinformatics Institute’s MetaboLights (Haug et al., 2013) databases that contained data from 64 and 85 studies, respectively. Query phenotypes may be selected from those already in MetaPhen or pasted into the web interface as a list of metabolites and their fold changes (or log transformations thereof). For user phenotypes, metabolite identifiers are recognized by first parsing them to remove chemical derivative or library-specific information and then mapping them to compound-specific IUPAC International Chemical Identifier (InChI) codes via a large database table mapping approximately 200,000 metabolite synonyms to over 8000 distinct metabolites. Most common metabolites will be recognized if a reasonably common name is used.

**Figure 1:**
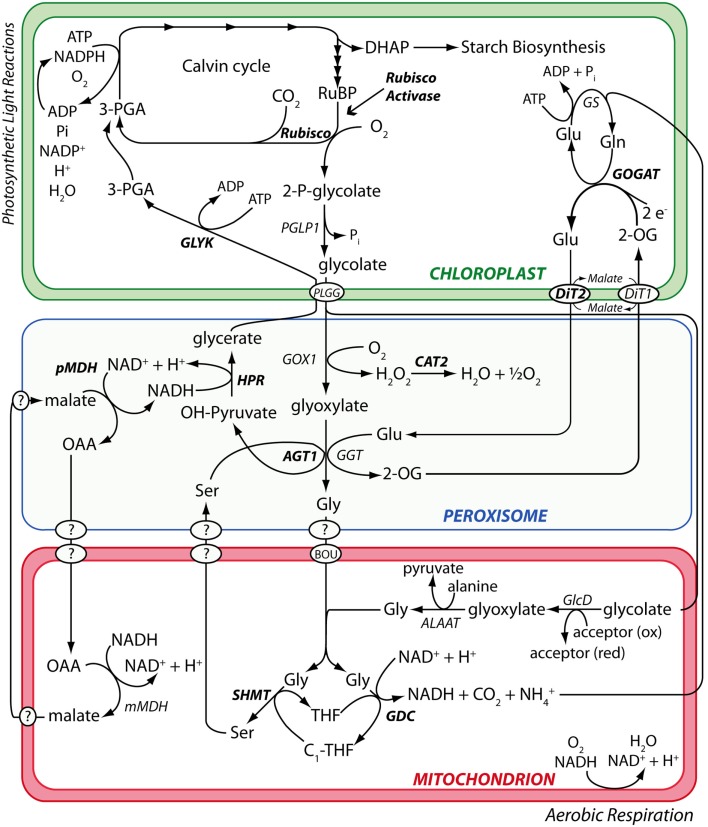
**The photorespiration metabolic pathway in *A. thaliana***. Metabolic phenotypes of *A. thaliana* mutants affected in various steps of the photorespiration metabolic pathway (highlighted in bold) were used to test the performance of the PhenoMeter. By correctly matching query phenotypes to their most functionally closely related counterparts (e.g., matching the phenotypes of mutants affected in the same gene rather than different genes), phenotypic pattern matching tools like the PhenoMeter should ideally discriminate between phenotypes of functionally closely related perturbations such as disruption at different steps of a metabolic pathway like photorespiration. Steps performed by unknown gene products are indicated with a “?.”

The primary dataset used to test the performance of candidate hit ranking statistics was a set of metabolic phenotypes obtained via untargeted GC/MS metabolomic analysis of polar leaf extracts of 26 *A. thaliana* metabolism mutants grown under a standard set of conditions. Thirteen of these (see Materials and Methods) had been previously characterized by others and shown to be impaired in enzymes of the photorespiration metabolic pathway (Figure 1). Our collection of known photorespiration mutants will hereon be referred to as the “reference mutants”. The remaining 13 metabolic phenotypes used in our performance test were of mutants isolated from an ethyl methane sulfonate (EMS)-mutagenized *A. thaliana* mutant population via a chlorophyll fluorescence-based forward genetic screen designed to detect photorespiration mutants (Badger et al., 2009).

Prior to this study, the new putative photorespiration mutants had not been characterized at the metabolic or genetic levels. However, it was expected that a high proportion of the new mutants would be impaired in the same metabolic enzyme as one or more of the reference mutants and therefore exhibit significantly similar metabolic phenotypes to those mutants. The rationale behind our performance test was that, after searching each of the new putative photorespiration mutant metabolic phenotypes against the reference phenotypes, the biochemical lesion indicated by the top match could be confirmed as correct or otherwise by the presence or absence of a mutation in the expected gene by next generation sequencing (NGS) or, if necessary, by the presence or absence of protein or enzymatic activity. Counting the percentage of correct top hits would then provide an assessment of each method’s performance. Below, we compare the performance of each ranking approach (see Table 1 for results).

**Table 1:** **Performance evaluation of four metabolic phenotypic similarity measures**.

			Top Ranking Hits Using Different Similarity Measures			
Query Mutant	Affected Enzyme	Lesion	*R*^2^	BWMC	FET2p	PM score
30-2A7	AGT1	G365R	***agt1-2***	***agt1-2***	*pmdh1pmdh2*	***agt1-2***
17-59G4	AGT1	L183F	***agt1-1***	***agt1-2***	***agt1-1***	***agt1-1***
32-34C7	AGT1	R30Q	***agt1-2***	*pmdh1pmdh2*	*glyk*	***agt1-2***
17-10F4	GLU1	G1252E	*dct*	***gls-113***	***gls-113***	***gls-113***
32-26C1	GLU1	R1306	***gls-113***	***gls-113***	***gls-113***	***gls-113***
24-27C2	GLU1	G104R	***gls-103***	*dct*	***gls-113***	***gls-113***
24-29A8	GLU1	P406L	***gls-103***	***gls-113***	***gls-113***	***gls-113***
24-2E5	GLU1	E579K	***gls-113***	***gls-113***	***gls-113***	***gls-113***
17-6E4	GLU1^a^	GLU1 Absent^a^	***gls-113***	***gls-103***	*dct*	***gls-113***
18-21E7	SHM1	E122K	***shm1***	*cat-2*	***mtkas-1***	***mtkas-1***
18-29A6	SHM1	E122K	***shm1***	***mtkas-1***	***mtkas-1***	***mtkas-1***
25-35D8	SHM1	R128H	***shm1***	***shm1***	***shm1***	***shm1***
24-14G7	MTKAS	G200R	***mtkas-1***	***mtkas-1***	***mtkas-1***	***mtkas-1***
		**% Correct:**	**92%**	**77%**	**77%**	**100%**

*Thirteen A. thaliana photorespiration mutant lines carrying severe non-synonymous mutations in conserved regions of known photorespiration genes (see “Affected enzyme” and “Lesion”) previously isolated by forward genetic screening in our lab (Badger et al., 2009) were genetically characterized by NGS-based genome resequencing (see Materials and Methods). The metabolic phenotypes of these “Query mutants” were analyzed by non-targeted GC/MS metabolomics and searched against the reference database of independently published “reference” photorespiration mutants using the PM. Four searches were performed with a different similarity measure being used to rank hits each time. The table shows the top hit returned for each query mutant using each similarity measure (correct hits are indicated in bold – note mtkas and shm1 phenotypes were treated as equivalent)*.

*^a^Absence of GLU1 confirmed by western blotting (Figure S1 in Supplementary Material) – it is not known whether other enzymes are also affected*.

### Candidate hit ranking statistic 1: *R*^2^

The first approach we tested was to simply rank hits according to *R*^2^ where *R* is the Pearson correlation coefficient between *X* and *Y*: equivalent equal length vectors representing the bait and prey metabolic phenotypes as treatment/control signal intensity ratios (SIRs or “fold-changes”) of the metabolites, transformed into so called “ResponseValues” (RVs) as follows:

$$\begin{array}{l} \text{Where SIR }>\text{ 1}:\text{ RV }=\text{ SIR }–\text{ 1}\\ \text{Where SIR }<\text{ 1}:\text{ RV }=\text{ 1 }-\;\left(\text{1}/\text{SIR}\right)\\ \text{Where SIR }=\text{ 1}:\text{ RV }=\;0 \end{array}$$

This transformation is similar to a log transformation in that it centers the data about 0 with increases, decreases, and non-responses being represented as positive, negative, and 0 values, respectively. However, unlike a log transformation, the RV transformation places the fold changes on a linear scale rather than a log scale and therefore does not down-weight stronger changes the way log transformation does. In all tests, a filter was applied to remove all metabolites with an absolute fold-change <1.5 (that is, a fold change between 1.5 and 1/1.5). This was to remove the influence of metabolites with marginal fold changes of questionable biological significance. Users may adjust this setting to suit their data.

This approach performed reasonably well in that, in 12 (92%) of 13 cases, the metabolic lesion indicated by the top match was confirmed either by the detection (by whole genome resequencing, see Materials and Methods) of a high-confidence and severe non-synonymous mutation in a conserved protein-coding region of the same gene as that affected in the top-matched reference mutant, or, in one case, by the detection of severe depletion of the relevant protein (Table 1). A single mismatch was obtained for the mutant, 17-10F4, that was matched to the chloroplast dicarboxylate transporter 2 (DIT2.1)-deficient reference mutant, *dct* (Somerville and Ogren, 1983). However, the genome re-sequencing-based detection of a high confidence mutation in the *GLU1* gene of 17-10F4 predicted a G1252E residue change in a conserved part of the GLU1 protein, thereby strongly supporting GLU1-deficient as the correct assignment (Table 1; Table S1 in Supplementary Material).

The enzymes, DIT2.1 and GLU1, are closely functionally connected (Figure 1) and the strongest metabolic changes observed in the DIT2.1- and GLU1-deficient reference mutants were similar (for example, 14- to 52-fold decreases in glutamate, ­aspartate, and serine and 4- to 14-fold increases in 2-­oxoglutarate). It is therefore not surprising that the *R*^2^ approach did not correctly resolve these phenotypes in every case, since *R*^2^ was strongly influenced by these larger fold changes. This sensitivity to larger fold changes raised concern that, by using *R*^2^ alone, useful discriminatory information contained within large numbers of small but nonetheless biologically meaningful metabolite responses could be effectively ignored if a relatively small number of metabolites changed strongly in similar ways in the query and reference phenotypes. Moreover, the *R*^2^ method would be prone to giving false negatives in instances where even a small number of large spurious metabolite responses are reported erroneously due to some overlooked technical issue (e.g., chemical contamination or peak integration error).

### Candidate hit ranking statistic 2: Biweight midcorrelation

Our concerns about the sensitivity of the *R*^2^ method to outliers and large fold changes led us to test the bicor function of the weighted correlation network analysis (WGCNA) *R* package (Langfelder and Horvath, 2008) as a potential alternative. This function performs a form of “robust” correlation known as “biweight midcorrelation” that is less sensitive to outliers than Pearson Correlation and has been used widely to measure similarity between gene expression profiles for the purposes of constructing gene co-expression networks (Langfelder and Horvath, 2012). Replacing *R*^2^ with the robust correlation coefficient calculated by the bicor function did indeed result in the previously mismatched phenotype of 17-10F4 being correctly top-matched to a GLU1-deficient reference mutant rather than the DIT2.1-deficient mutant (as observed using *R*^2^, see above). However, the appearance of a number of new mismatches brought the success rate of this approach down to only 10 (77%) out of 13 (Table 1) suggesting a decrease in overall performance relative to the *R*^2^ and hence that down-weighting of higher fold changes with larger absolute deviations did not enhance discrimination. This method was therefore not tested further and will be referred to as the “BWMC” method. For mathematical details of BWMC, please refer to Langfelder and Horvath (2012).

### Candidate ranking statistic 3: Fisher’s exact test for response overlap (FET2p)

The observation of incorrect top matches with the correlation-based ranking methods led us to question whether better performance might be gained by considering only directions of metabolite changes rather than their fold-changes. To this end, we tried ranking phenotypic similarities according to the “qualitative overlap” of the two metabolic phenotypes as determined by counting: (a) the number of metabolites that are increased in both phenotypes; (b) the number of metabolites that are decreased in both phenotypes; (c) the number of metabolites that are increased in the query but decreased in the reference; and (d) the number of metabolites that are decreased in the query but increased in the reference; and using these as input into a two-tailed Fisher’s Exact Test (Fisher, 1922). The *p*-value obtained by this method will be referred to as “FET2p.”

Ranking hits in order of FET2p resulted in 10 (77%) of the 13 mutants being correctly top-matched (Table 1). Using this method eliminated the mismatching of the new *shm1* mutant 18-21E7 to the *cat-2* reference that was observed using the *R*^2^ and BWMC methods and mismatches of the new *gls* (GLU1-impaired) mutants, 17-10F4 and 24-27C2, to the *dct* reference as observed using *R*^2^ and BWMC, respectively. This suggested that qualitative (directional) aspects these metabolic phenotypes, exposed by removing the contribution of fold change magnitudes to the ranking, contained important discriminatory information not sufficiently captured by *R*^2^ or BWMC. The FET2p method, however, produced new mismatches for two mutants identified by genome resequencing as defective in peroxisomal serine/alanine: glyoxylate aminotransferase 1 (AGT1) on the basis of severe mutations in the *AGT1* gene (Table S1 in Supplementary Material). One of these (30-2A7) was mismatched to the *pmdh1pmdh2* reference mutant impaired in peroxisomal malate dehydrogenase (pMDH) activity metabolically closely connected to AGT1 in the peroxisome. The other (32-34C7) was mismatched to the *glyk* reference mutant defective in the plastidic enzyme, glycerate kinase (GLYK), which functions just downstream of AGT1 in the photorespiratory pathway. Importantly, these mutants were both correctly top-matched to AGT1-deficient reference phenotypes using the *R*^2^ method (Table 1), suggesting that metabolite fold changes contained information necessary to discriminate the qualitatively similar AGT1-, pMDH-, and GLYK-deficient phenotypes. Another mutant (17-6E4) correctly matched to GLU1-deficient *gls* reference mutants using *R*^2^ and BWMC and confirmed to be deficient in GLU1 protein by western blotting (Figure S1 in Supplementary Material), was mismatched to *dct* using the FET2p method.

### Candidate ranking statistic 4: *R*^2^ × (−log_10_(FET2p))

The fact that that the *R*^2^ and FET2p methods yielded complementary sets of correct top matches suggested that they were sensitive to complementary types of functional discriminatory information within metabolic phenotypes. Based on this observation, we hypothesized that a hybrid scoring function allowing both metrics to contribute to rankings might outperform either metric alone. We therefore tested the ability of the formula *R*^2^ × (−log_10_(FET2p)) to yield correct top matches for the 13 mutants. Indeed, we found that this approach outperformed all the previous metrics, yielding correct top matches in all 13 (100%) of the test mutants (Tables 1 and 2). We therefore adopted this formula as the implemented hit ranking metric.

$$\text{PhenoMeter Hit Ranking Metric }=\;R^{2}\text{ × }(-{\text{log}}_{10}(\text{FET2p}))$$

**Table 2:** **The PM score gave correct top matches in all test cases**.

Query^a^	Top hit^b^	FET2p	*R*	PM score	*p*_non-bio_
*32-26C1*	*gls1-113*	2.4E-13	0.88	9.7	*p* < 10^−308^
*17-10F4*	*gls1-113*	1.5E-19	0.67	8.4	*p* < 10^−308^
*25-35D8*	*shm1*	8.6E-13	0.73	6.4	*p* < 10^−308^
*24-29A8*	*gls1-113*	1.3E-12	0.72	6.2	*p* < 10^−308^
*17-6E4^c^*	*gls1-103*	2.1E-07	0.93	5.8	*p* = 6.7E^−195^
*24-14G7*	*mtkas-1*	2.9E-09	0.71	4.3	*p* < 10^−308^
*24-2E5*	*gls1-113*	4.3E-08	0.63	3.0	*p* < 10^−308^
*18-29A6*	*mtkas-1*	1.5E-14	0.40	2.3	*p* < 10^−308^
*18-21E7*	*shm1*	7.9E-07	0.5	1.6	*p* < 10^−308^
*17-59G4*	*agt1-1*	1.0E-03	0.66	1.3	*p* < 10^−308^
*30-2A7*	*agt1-2*	7.2E-03	0.65	0.9	*p* < 10^−308^
*32-34C7*	*agt1-2*	4.8E-03	0.55	0.7	*p* < 10^−308^
*24-27C2*	*gls1-113*	2.0E-09	0.15	0.2	*p* < 10^−308^

*^a^ID of the sequenced independent photorespiratory mutant whose metabolic phenotype was used as query*.

*^b^ID of the known reference photorespiratory mutant whose phenotype gave the highest PM score. All hits were determined to be correct by the detection in the test mutants (by NGS) of severe non-synonymous mutations in conserved protein coding regions of the same genes as indicated by the hit mutant*.

*^c^The hit returned by 17-6E4 was confirmed as correct by western blotting which showed the absence of GLU1 protein (Figure S1 in Supplementary Material)*.

### The phenometer score

The *R*^2^ × (−log_10_(FET2p)) formula used to rank hits responds only of the strength of the similarity between the query and reference phenotypes, returning a positive value regardless of whether a match is positively or negatively correlated with the query. While this property made the formula ideal for ranking hits, we also needed a metric that would indicate not only the strength but also the direction of the relationship. To this end, we adapted the formula by multiplying it by sgn(*R*) so that the sign of the score would be the same as the sign of the correlation between the query and reference phenotypes (sgn is a mathematical function that extracts the sign of its parameter by outputting −1 when it is negative, +1 when it is positive and 0 when it is equal to 0):

$$\text{PhenoMeter Score = sgn}\left(R\right)\text{ × }R^{2}\text{ × }(-{\text{log}}_{10}(\text{FET2p}))$$

**Figure 2:**
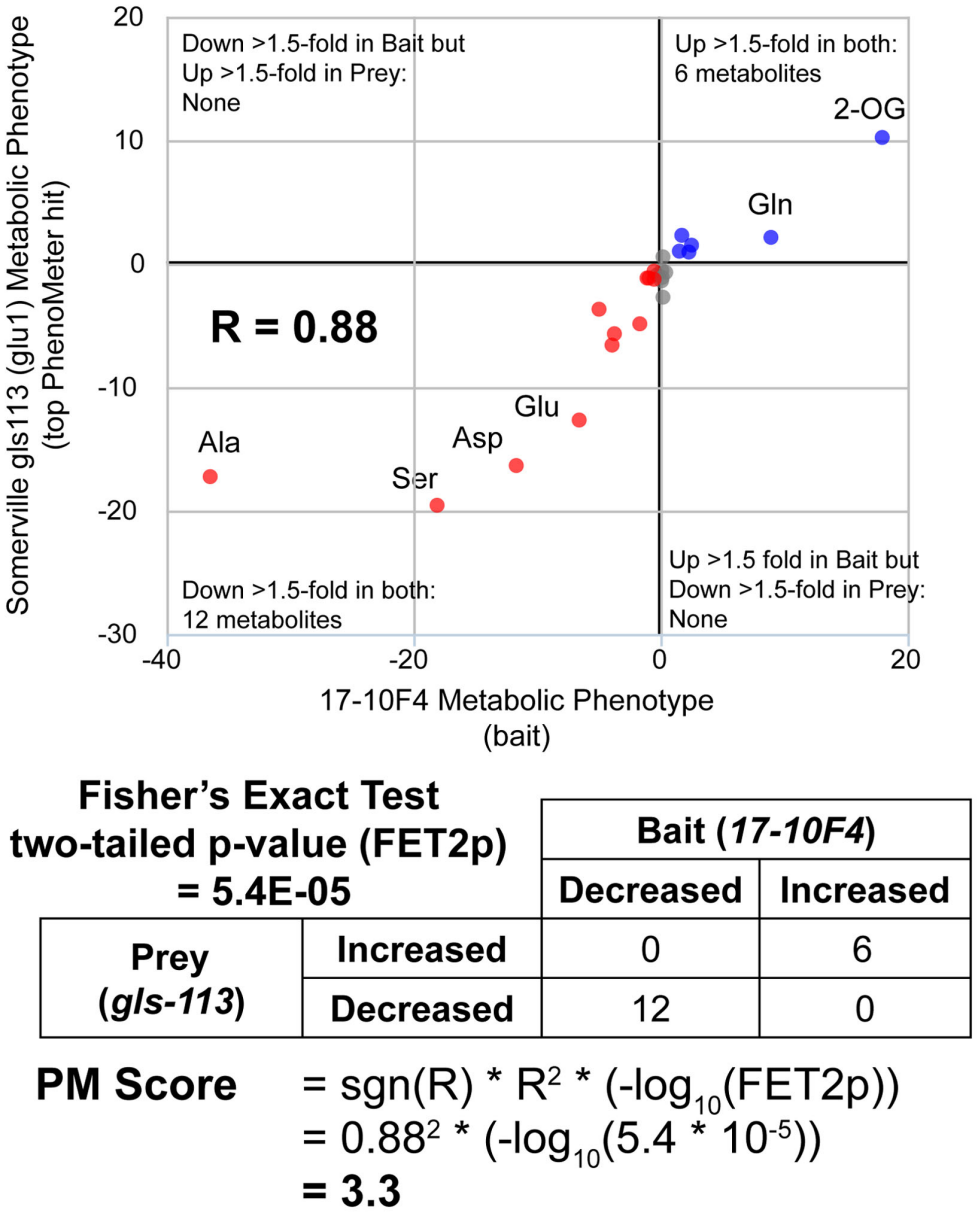
**Calculation of PhenoMeter (PM) score**. The procedure for calculating the PhenoMeter similarity score consists of several stages. First, metabolites that are not represented or do not increase or decrease by at least the minimum threshold (1.5-fold by default) in both bait and prey are discarded. Then, signal intensity ratios (SIRs) associated with each metabolite are transformed to ResponseValues (RV = SIR–1 where SIR > 1 and RV = (−1/SIR) + 1 where SIR <1). The correlation between the RVs of the bait and prey phenotypes is then calculated. The PhenoMeter then counts the number of metabolites that are (1) increased above threshold in both phenotypes; (2) decreased below threshold in both phenotypes; (3) decreased below threshold in the bait but increased above threshold in the reference; and (4) increased above threshold in bait but decreased below threshold in reference; and then uses these values as input into a two-tailed Fisher’s Exact Test to calculate the statistical significance of the qualitative overlap of the two phenotypes (FET2p). The PM score is then calculated using the formula PM score = sgn(*R*)*R^2^*(–log_10_(FET2p)).

While simply replacing *R*^2^ with *R* would have achieved the same sign-changing effect, we observed lower matching performance when *R*^2^ was replaced with *R* (data not shown due to limited space). The fact that the magnitude of the PM score is derived from two readily understood statistics (*R*^2^ and FET2p) makes its “strength” readily interpretable in familiar statistical terms. For example, the fact that a match with a marginally significant FET2p of 0.05 and an *R*^2^ of 0.8 (an above average *R*^2^ for genuine matches between functionally equivalent phenotypes; see Table 2) would have a PM score of ~1 (1.040824) makes this score a useful benchmark since scores <1 must either have low *R*^2^ or insignificant FET2p while scores >>1 must at least have a highly significant FET2p if not a high *R*^2^ as well. Thus, as a “rule of thumb,” scores <1 may be considered ‘weak’ while scores >>1 may be considered “strong.” An example PM score calculation is illustrated in Figure 2.

### Permutation-based estimation of statistical significance: *p*_non-bio_

An important issue associated with the use of pattern matching tools employing custom scoring methods is that of assessing statistical significance. An intuitive way of assessing the significance of a hit with a particular score is to estimate the probability of it arising by chance from a query that is the same in every way except that the associations between fold-change values and metabolite labels have been randomized to destroy the biological meaningfulness of the query pattern. After all, under the null hypothesis that the query was biologically meaningless, shuffling the metabolite labels should have no significant effect on the magnitude of the returned score. Thus, adopting the traditional alpha cut-off of 0.05, we may consider a significant hit as one with a score expected to be exceeded in magnitude in fewer than 5% of cases when metabolite shuffling and searching is repeated many times.

Theoretically, the probability of obtaining a given score after metabolite labels have been reshuffled is sensitive to a variety of search parameters:

1. The size of the search space. The larger the number of reference phenotypes in the search space, the greater the probability of getting at least one high score by chance.
2. The number of metabolites in the query. As the number of metabolites compared is increased, the lowest achievable *p*-value from the Fisher’s Exact Test is decreased (and the potential magnitude of the PM score increased).
3. The identities of metabolites in the query. Some metabolites tend to be more biologically responsive than others and thus have greater potential to contribute to higher *R*^2^ values.
4. The distribution of fold changes in the query (and hence also the fold-change filter threshold setting) could affect the capacity to achieve high *R*^2^ values.

**Figure 3:**
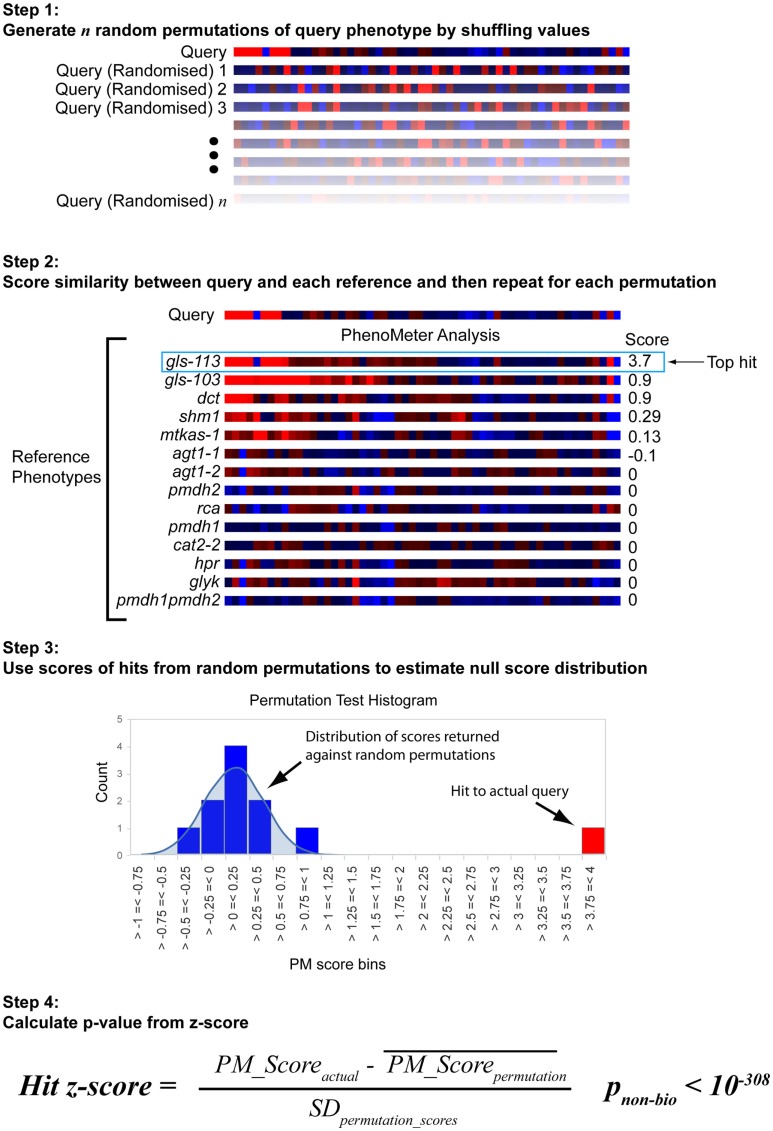
**Estimation of statistical significance via permutation testing**. The probability of obtaining a given PhenoMeter score by chance is dependent upon algorithm settings and the nature of the bait and reference phenotype search space. To estimate the chance of obtaining the reported score by chance, each bait search is therefore accompanied by a permutation test in which random permutations of the bait phenotype are searched in an otherwise identical manner. The mean and standard deviation (SD) of the scores from these searches is then used to calculate a *z*-score for the actual score from which a *p*-value is estimated. We call this *p*-value *p*_non-bio_ because it represents the probability of the match score not having arisen out of biology.

The approach used to estimate the probability of a hit with a given score being a false positive under the null hypothesis is shown in Figure 3. Each search begins by generating 30 permutations of the original query in which the metabolite labels are randomly shuffled. These are then searched against the chosen reference phenotype set in exactly the same way as the original query. The null score distribution is then modeled as a normal distribution from the mean and SD of all the scores returned by the permutated phenotypes (the total number of “null” scores being equal to 30 times the number of phenotypes in the search space).

**Figure 4:**
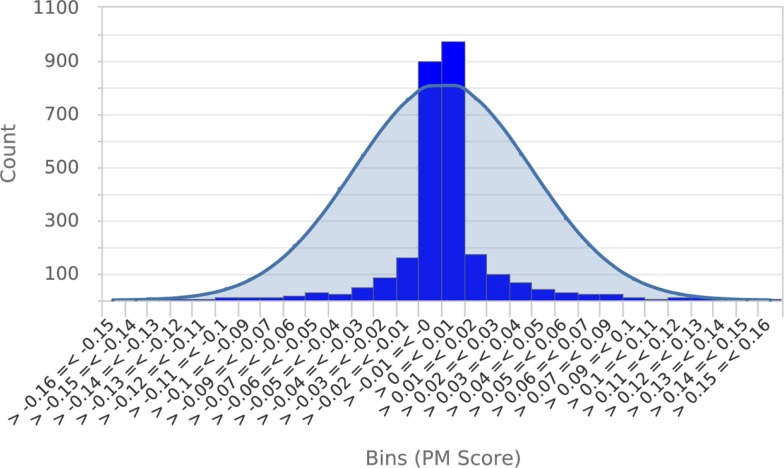
**Null score distribution associated with 500 permutations of a typical query**. To establish that the normal distribution was an appropriate model of the typical null score distribution, a histogram of scores obtained from 500 permutations of a typical query phenotype was prepared and overlaid with a plot of the normal distribution calculated from the mean and SD of scores. This clearly shows that the normal distribution is a conservative model of the null score distribution. Essentially identical results were obtained from permutations of four other metabolic phenotypes.

To establish that a normal distribution was a reasonable model of the typical null score distribution, we permuted the phenotype of five different mutants 500 times each, prepared histograms of the resulting null PM hit scores and overlaid them with plots of the normal distribution modeled from the means and SDs of the null scores (see Figure 4 for a representative example). These results clearly established that the normal distribution was in fact a conservative model of the empirical null distribution. Conveniently, this made it appropriate to use the common *z*-statistic to estimate the percentage of random permutations that would be expected to exceed any given score:

$$\text{Hit }z-\text{score}=\frac{\text{PM}\_{\text{Score}}_{\text{actual}}\text{- }\bar{{\text{PM\_Score}}_{\text{perm}}}}{{\text{σ}}_{\text{perm scores}}}$$

where PM_Score_actual_ is the score of the original unshuffled phenotype match and $\bar{{\text{PM\_Score}}_{\text{perm}}}$ and σ_perm scores_ are the mean and SD of the PM scores returned from the permuted queries, respectively.

The *p*-value derived through the above approach treats the fold changes of different metabolites as independent observations. For the purposes of testing the null hypothesis defined earlier, this assumption of independence is appropriate given that different metabolites are generally detected as independent instrument signals whose magnitudes bear no significant influence on one another and should exhibit no significant correlations attributable to systematic technical variation provided that effective steps were taken to avoid such systematic technical variation (e.g., block randomization of analytical batch sequences). Thus, as long as the above technical requirements are met, this *p*-value can be interpreted as an estimate of the probability that the observed match score *did not* emerge out of biology. We therefore refer to this value as *p*_non-bio_. It is important to note that the *biological* non-independence of metabolites which arises from metabolic network structure is not relevant to the hypothesis outlined above.

### Speed performance

For phenotypic similarity searches to become a practical part of daily operations, results need to be returned within seconds or minutes rather than hours or days. Computational efficiency was therefore another factor behind the decision to use simple statistics as the basis of phenotypic similarity measurement. To evaluate speed performance of the PM, we ran some different kinds of searches and recorded the completion times (Table 3). From these results, it can be seen that search times associated with typical usage cases range from under 2 s for simple searches with less than 100 metabolites to 10 min for more complex searches including thousands of metabolite signals.

**Table 3:** **Completion times associated with typical PhenoMeter usage cases**.

Description	Number of query phenotypes	Number of reference phenotypes	Unknown metabolites included (Y|N)	Completion time (s)
Search phenotype against target reference phenotypes for classification	1	14	N	1.9
Search phenotype against target reference phenotypes for classification	1	14	Y	3.7
Untargeted search of single phenotype against entire database for non-biased annotation	1	442	Y	35
Search phenotypes against themselves to construct a similarity network	36	36	N	304
Search phenotypes against themselves to construct a similarity network	36	36	Y	589

### Visualization of phenotypic similarity networks

In the validation experiments described earlier, we observed that *A. thaliana* mutants affected in different but functionally connected enzymes displayed similar metabolic phenotypes. Indeed, the mirroring of gene–gene functional connections in phenotype–phenotype similarities has also been observed in yeast (Fraser and Plotkin, 2007; McGary et al., 2007), nematodes (Lee et al., 2008), and plants (Messerli et al., 2007; Fukushima et al., 2014). The observation that human diseases associated with similar phenotypes are frequently mediated by genes that are connected via functional modules such as pathways or ­protein–protein interaction networks (Lage et al., 2007) has led to the concept of the “modular nature of human genetic disease” (Oti et al., 2008). This correlation between functional genetic relationships and phenotypic relationships has been exploited successfully in the prediction of the disease-causing roles of genes from functional genomics data (Wu et al., 2008).

**Figure 5:**
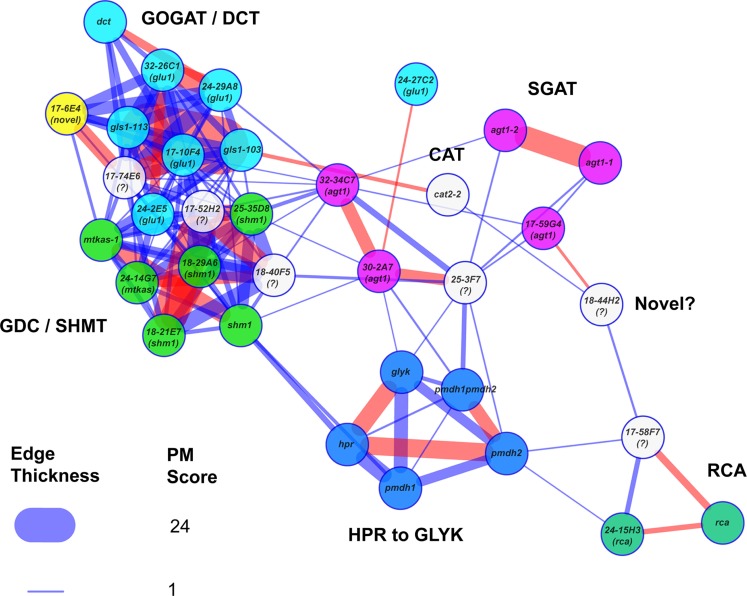
**Metabolic phenotype similarity networks reveal functional communities**. The known reference and new photorespiratory mutants were selected as both baits and potential prey in a PhenoMeter search to generate a similarity network with a force directed layout. To help reveal clustering within the network, weak matches (edges) with FET2p > 0.007 or *R*^2^ < 0.09 were filtered out to leave only moderate to strong matches. To highlight the fact that network structure reflected functional links between the mutants, mutants within various functional categories (labeled) were highlighted in the same color. Stronger similarities between mutant phenotypes (higher PM score) are represented by thicker edges. Edges in red represent the top hit of at least one of the nodes. Mutants marked with a “?” were not included in the set of mutants that were checked for mutations by next generation sequencing. The loose connectivity of *18-44H2* with the rest of the network and the fact that its causative mutation was mapped to a region free of known photorespiratory genes (data not shown) suggests it might be a novel class of photorespiratory mutant. The novel *glu1-like* mutant (*17-6E4*), highlighted in yellow, was tightly connected within the GOGAT/DCT neighborhood despite having no consequential mutations in any known photorespiratory genes.

The PM provides an opportunity to explore the structures of metabolic phenotypic similarity networks and obtain insight into the functional interaction networks that underlie them. To this end, we incorporated network graph visualization of metabolic phenotypic similarity networks into the PM. As a demonstration, we generated a network graph of the metabolic phenotypes of the *A. thaliana* photorespiration mutants used to develop the PM similarity scoring method. We then colored the nodes according to pathway function to visually explore whether network modules reflected metabolic functional modules (Figure 5). Mutants affected in Fd-GOGAT (GLU1) were co-located (in the upper left of the graph) with the *dct* mutant defective in the closely functionally connected dicarboxylate transporter (DiT2.1). This cluster was highly interconnected with another cluster (lower left) made up of mutants affected in two other closely metabolically linked enzymes: glycine decarboxylase (GDC) and serine hydroxymethyltransferase (SHMT). This interconnection reflects the shared involvement of GLU1, DiT2.1, GDC, and SHMT in the photorespiratory nitrogen cycle (Figure 1). To the bottom center of the graph was a cluster made up of mutants affected in enzymes hydroxypyruvate reductase (HPR), pMDH, and GLYK that function together in pathway converting hydroxypyruvate to 3-phosphoglycerate. Consistent with the notion that phenotypic similarity networks reflect functional networks, the *agt1* mutants affected in AGT1 – an enzyme that links HPR to the photorespiratory nitrogen cycle through the transamination of glyoxylate using serine as an amino donor – were connected with both the HPR/pMDH/GLYK cluster and the cluster made up of mutants affected in the photorespiratory nitrogen recycling enzymes GLU1, DiT2.1, GDC, and SHMT.

### Cross-study metabolic phenotype matching

To investigate the potential for cross-study phenotype matching with the PM, we tested whether independently published metabolic phenotypes for various photorespiration mutants transferred from non-photorespiratory high CO_2_ to photorespiratory air conditions for various durations against our reference set (Table 4). We first tested whether the phenotypes of mutants impaired in mitochondrial glycine to serine conversion could be correctly top-matched to our reference *shm1* and *mtkas-1* photorespiration mutants impaired in the same process. These mutants exhibit strong metabolic phenotypes and their comparison in the same species therefore represents a best-case scenario for achieving significant matches. Indeed, phenotypes reported for *shm1* by Collakova et al. (2008) and Eisenhut et al. (2013) both returned significant top matches to either our *shm1* or *mtkas-1* reference despite considerable differences in air treatment time (Table 4). Similar results were obtained (Table 4) for the phenotypes of other mutants affected glycine-to-serine conversion – namely the *purU* mutant defective in 10-formyl tetrahydrofolate (THF) deformylase activity required to prevent accumulation of 5-formyl-THF that potently inhibits GDC/SHMT activity in the mitochondrial matrix (Collakova et al., 2008) and the *bou-2* mutant defective in the putative mitochondrial glycine transporter, A BOUT DE SOUFFLE, presumably required to transport glycine into mitochondria so it can be converted into serine (Eisenhut et al., 2013).

**Table 4:** ***PhenoMeter* analyses of previously published metabolic phenotypes**.

Study	Mutant	Time in air	Top hit	FET2p	*R*	PM score	*p*_non-bio_
Collakova et al. (2008)	*shm1*	3 d	*mtkas-1*	0.28	0.6	0.21	3.1E−4
	*purU* dKO	3 d	*shm1*	0.008	0.8	1.47	9E−130
Eisenhut et al. (2013)	*shm1*	17 h	*shm1-1*	0.069	0.5	0.28	1E−9
	*bou-2*	17 h	*mtkas-1*	0.03	0.5	0.35	10E−10
Pérez-Delgado et al. (2013)	*Ljgs2-2*	2 d	*shm1*	0.06	0.76	0.69	9E−67
	*Ljgs2-2*	3 d	*pmdh1pmdh2*	0.08	0.25	0.07	0.04
	*Ljgs2-2*	4 d	*shm1*	0.3	0.67	0.24	1E−15
	*Ljgs2-2*	6 d	*pmdh1pmdh2*	0.06	0.56	0.38	5.6E−19
	*Ljgs2-2*	8 d	*pmdh1pmdh2*	0.1	0.48	0.2	1E−7
	*Ljgs2-2*	10 d	*No hits*				
Timm et al. (2012)	*hpr1*	1 d	*shm1*	0.2	0.48	0.16	5.5E−4
	*hpr1*	3 d	*pmdh2*	0.2	0.49	0.15	3E−4
	*hpr1*	5 d	*pmdh2*	0.17	0.37	0.11	5E−2
	*pglp1*	1 d	*pmdh2*	0.24	0.54	0.18	3.5E−3
	*pglp1*	3 d	*hpr1*	0.024	0.58	0.54	5E−21
	*pglp1*	5 d	*pmdh2*	0.012	0.52	0.52	6E−25

*Selected metabolic phenotypes previously reported for *Arabidopsis thaliana* photorespiratory mutants following transfer from non-photorespiratory high CO_2_ conditions to air for various times were submitted as queries to the *PhenoMeter* using the reference photorespiratory mutant set as the search space*.

We next tried searching phenotypes reported for *Ljgs2-2*, a *Lotus japonicus* photorespiration mutant affected in plastidic glutamine synthetase (GS) (Pérez-Delgado et al., 2013). These phenotypes were strong but our reference set did not include any *A. thaliana* or *L. japonicas* GS mutants so we wanted to test whether the phenotypes of *Ljgs2-2* had enough similarity to our reference phenotypes to yield hits consistent with its photorespiratory impairment. Indeed, significant matches to our reference phenotypes were returned, the highest scoring ones being to *shm1* and *pmdh1pmdh2* (Table 4). Moreover, an unbiased search of the entire database, which contains hundreds of phenotypes besides those of photorespiration mutants, returned a set of 27 significant hits of which 19 (70%), including the top two (*p*_non-bio_ = 4E−281 and 7E−81), were associated with photorespiration mutants despite photorespiration mutant phenotypes comprising only 4% of the phenotypes in the database.

As a further challenge, we tried searching phenotypes reported for *hpr1* and *pglp1* (Timm et al., 2012). The *hpr1* mutant affected in peroxisomal hydroxypyruvate reductase 1 (HPR1) and a number of phenotypically similar *pmdh* mutants affected in one of its metabolic partner enzymes, pMDH, were represented in our reference set. However, the metabolic phenotype of *hpr1* is mild and reportedly quite dependent on the length of time exposed to air (Timm et al., 2012), suggesting that technical noise and the influences of experimental differences might preclude matching. Nonetheless, *hpr1* phenotypes recorded after 1, 3, and 5 days of air treatment gave significant hits to *shm1*, *pmdh2*, and *pmdh2*, respectively, albeit with low scores (Table 4). Given the functional and phenotypic relatedness of *hpr1* and *pmdh* mutants, it is not surprising that *hpr1* was mismatched to *pmdh2*. The mismatch to *shm1* over *hpr1* or *pmdh* references was more surprising but nonetheless significant.

Our reference set did not include any reference phenotypes for phosphoglycolate phosphatase mutants. However, the phenotypes reported for *pglp1* exposed to air for 1, 3, and 5 days gave significant matches to *pmdh2*, *hpr1*, and *pmdh2* with *p*_non-bio_ values of 3.5E−3, 5E−21, and 6E−25, respectively (Table 4). Searching the five-day phenotype against the entire database confirmed that *pmdh2* and *hpr1* were still returned as the two most significant hits.

### Examples of matching responses to non-photorespiratory genetic perturbations

The PM approach is not only applicable to phenotypes of photorespiratory mutants but also to all kinds of metabolic responses. To demonstrate this, we tested whether searching the metabolic phenotypes of mutants affected in genes outside the photorespiration pathway would also return hits to functionally related mutants (Table 5). First, we searched the entire database using the metabolic phenotype of the *A. thaliana alx8* mutant – an EMS mutant in a Col-0 ecotype background that harbors a loss-of-function mutation in the gene, At5g63980, encoding the bi-functional SAL1/FRY1/RON1 nucleotidase/phosphatase shown to be a negative regulator of abiotic stress defense responses linked to chloroplast retrograde signaling (Wilson et al., 2009; Estavillo et al., 2011). The top hit (PM score = 7.35; Table 5) was to the phenotype of *fry1-1*, a functionally equivalent *A. thaliana* mutant (Wilson et al., 2009) harboring an independent loss-of-function mutation in the same gene in a different wild-type background (C24). This was the only hit with a PM score >1, consistent with the fact that the *fry1-1* phenotype was the only functionally similar phenotype in the database.

**Table 5:** **Matching metabolic phenotypes of functionally linked *A. thaliana* metabolism mutants affected outside the photorespiration pathway**.

Query	Top hit	PM score	Functional link
*A. thaliana* *alx8* SAL1/FRY1/RON1 nucleotidase/phosphatase loss-of-function mutant (Wilson et al., 2009)	*A. thaliana* *fry1-1* SAL1/FRY1/RON1 nucleotidase/phosphatase loss-of-function mutant (Wilson et al., 2009)	7.35	Independent functionally equivalent mutation, different genetic background
*A. thaliana* *aox1a-1* mitochondrial alternative oxidase 1a T-DNA insertion gene knockout mutant under combined drought and moderate high light stress (Giraud et al., 2008)	*A. thaliana* *aox1a-2* mitochondrial alternative oxidase 1a T-DNA insertion gene knockout mutant under combined drought and moderate high light (Giraud et al., 2008)	10.22	Independent functionally equivalent gene knockout
*A. thaliana* *4cl1-1* 4-coumarate CoA ligase 1 T-DNA insertion gene knockout mutant (Vanholme et al., 2012)	*A. thaliana* *4cl1-2* 4-coumarate CoA ligase 1 T-DNA insertion gene knockout mutant (Vanholme et al., 2012)	4.17	Independent functionally equivalent gene knockout
*A. thaliana* *4cl2-1* 4-coumarate CoA ligase 2 T-DNA insertion gene knockout mutant (Vanholme et al., 2012)	*A. thaliana* *4cl2-3* 4-coumarate CoA ligase 2 T-DNA insertion gene knockout mutant (Vanholme et al., 2012)	0.95	Independent functionally equivalent gene knockout
*A. thaliana* *c4h-2* cinnamate 4-hydroxylase T-DNA insertion gene knockout mutant (Vanholme et al., 2012)	*A. thaliana* *c4h-3* cinnamate 4-hydroxylase T-DNA insertion gene knockout mutant (Vanholme et al., 2012)	1.42	Independent functionally equivalent gene knockout
*A. thaliana* *ccaomt1-3* caffeoyl-CoA O-methyltransferase 1 T-DNA insertion gene knockout mutant (Vanholme et al., 2012)	*A. thaliana* *ccaomt1-5* caffeoyl-CoA O-methyltransferase 1 T-DNA insertion gene knockout mutant (Vanholme et al., 2012)	5.08	Independent functionally equivalent gene knockout
*A. thaliana* *pal1-2* phenylalanine ammonia lyase 1 T-DNA insertion gene knockout mutant (Vanholme et al., 2012)	*A. thaliana* *pal1-3* phenylalanine ammonia lyase 1 T-DNA insertion gene knockout mutant (Vanholme et al., 2012)	0.74	Independent functionally equivalent gene knockout
*A. thaliana* *comt-1* caffeic acid O-methyltransferase T-DNA insertion gene knockout mutant (Vanholme et al., 2012)	*A. thaliana* *f5h1-2* ferulate 5-hydroxylase 1 T-DNA insertion gene knockout mutant (Vanholme et al., 2012)	1.65	Functionally linked gene knockout. The metabolic product of COMT is the substrate of F5H1.

Next, we performed a similar search using the metabolic phenotype of the *A. thaliana* mutant *aox1a-1*, a SALK T-DNA insertion gene knockout mutant line lacking a functional copy of the *ALTERNATIVE OXIDASE 1A* gene (*AOX1A*; At3g22370) relative to wild-type control when both genotypes have been treated together with combined drought and moderate high light stress (Giraud et al., 2008). The top hit was to the metabolic phenotype of *aox1a-2* – an independent *AOX1a* T-DNA knockout mutant from the SAIL collection – under the same conditions (PM score = 10.22; Table 5).

A recent systematic study of *A. thaliana* lignin biosynthesis mutants originating from outside our group (Vanholme et al., 2012) provided a useful set of high-quality metabolic phenotypes for independent knockout mutant pairs affected in the same genes by independent insertions. We searched the reported metabolic phenotypes of the following mutants from that study against the entire database to test whether they would each return the functionally equivalent independent knockout as the top hit: *4cl1-1* (4-coumarate CoA ligase 1 knockout), *4cl2-1* (4-coumarate CoA ligase 2 knockout), *c4h-2* (cinnamate 4-hydroxylase knockout), *ccaomt1-3* (caffeoyl CoA O-methyltransferase 1 knockout), *pal1-2* (phenylalanine ammonia lyase 1 knockout), and *comt-1* (caffeic acid O-methyltransferase knockout). In five (83%) of these six cases, the top hit was to an independent mutant affected in exactly the same gene (PM scores ranging from 0.74 to 5.08; Table 5). The one exception was for the *comt-1* bait phenotype for which the top hit (PM score = 1.65) was to the phenotype of the closely functionally linked *f5h1-2* (ferulate 5-hydroxylase 1 knockout) mutant (the metabolic product of COMT is the substrate of F5H1 in the lignin biosynthesis pathway). Overall, the above results confirm that the PM approach is generally applicable to all kinds of mutants having characteristic metabolic phenotypes.

### Examples of matching responses to environmental and biochemical perturbation

Thus far, we have only demonstrated the PM matching of metabolic phenotypes associated with genetic perturbation. However, the PM approach could also be used to detect functional links between metabolic responses to other forms of perturbation such as environmental or biochemical perturbation. To demonstrate this, we searched metabolic responses to such perturbations against the database in cases where responses to functionally similar perturbations were present in the database (Table 6).

**Table 6:** **Matching metabolic responses to functionally linked chemical and environmental perturbations**.

Query	Top hit	PM score	Functional link
Response of *A. thaliana* cells to 16 h of respiratory inhibition with rotenone (Garmier et al., 2008)	Response of *A. thaliana* cells to 12 h of respiratory inhibition with rotenone (Garmier et al., 2008)	6.7	Same treatment for different durations
Response of germinating *Oryza sativa* seedlings to 24 h of anoxic treatment after 24 h of aerobic germination (Narsai et al., 2009)	Response of germinating *Oryza sativa* seedlings to 48 h of constitutive anoxic germination (Narsai et al., 2009)	12.5	Same treatment for different durations and at slightly different developmental stages
Response of *L. japonicus* shoots to 150 mM NaCl stress (Sanchez et al., 2008)	Response of *L. japonicus* shoots to 100 mM NaCl stress (Sanchez et al., 2008)	9.6	Same treatment with different doses and durations
Response of *A. thaliana* seedlings to 13 days of germination under constitutive sulfur deficiency (Nikiforova et al., 2005)	Response of *A. thaliana* seedlings to 10 days of germination under constitutive sulfur deficiency (Nikiforova et al., 2005)	1.8	Same treatment for different durations
Response of *A. thaliana* seedlings to 24 h of cold stress (Kaplan et al., 2004)	Response of *A. thaliana* seedlings to 48 h of cold stress (Kaplan et al., 2004)	12.9	Same treatment for different durations
Response of *A. thaliana* seedlings to 120 min of heat stress (Kaplan et al., 2004)	Response of *A. thaliana* seedlings to 60 min of heat stress (Kaplan et al., 2004)	1.92	Same treatment for different durations

The first response we searched was that of *A. thaliana* cells to 16 h of treatment with rotenone – a pharmacological inhibitor of mitochondrial electron transport chain complex I (Garmier et al., 2008). The top hit (PM score = 6.7) was to the response of the same cells to the same treatment for 12 h (the previous time point in the same rotenone treatment time course). Similarly, the top hit (PM score = 12.5) to the response of germinating *Oryza sativa* (rice) seedlings to 24 h of anaerobic treatment after 24 h of aerobic germination (relative to a constitutive aerobic germination control) was the response of rice seedlings to 48 h of constitutive anaerobic germination (relative to an aerobically germinated control).

To demonstrate that such matching between related responses was possible for responses reported by other authors and for responses to treatments other than respiratory perturbation, we also searched the responses of *Lotus japonicus* to salt stress (Sanchez et al., 2008) and *A. thaliana* to sulfur deficiency (Nikiforova et al., 2005) and cold stress (Kaplan et al., 2004), and heat stress (Kaplan et al., 2004). In each case, the top hit was to the response recorded for the same treatment for a different duration or dosage in the same time course or dosage series (Table 6).

As an additional validation, we constructed a network from the metabolic responses of *A. thaliana* plants to various durations of 4°C cold stress (1, 4, 12, 24, 48, and 96 h) and 40°C heat stress (5, 15, 30, 60, 120, and 240 min) previously reported by Kaplan et al. (2004) in a time course metabolomic study (Figure S2 in Supplementary Material). Nodes corresponding to cold stress responses formed a tightly connected cluster that was, in turn, loosely connected to a more sparsely connected cluster corresponding to the heat stress responses. Within each cluster, the strengths of PM scores between nodes reflected the temporal order of the associated time course. That is, beginning from the earliest time-point, following the pathway of highest PM scores from node to node (without backtracking) correctly traced out the temporal pathway from the start to the end of the relevant time course. This confirms that the metabolic responses of *A. thaliana* to the temperature stresses follow orderly progressions through time [as originally demonstrated by Kaplan et al. (2004)] and that this progression is accurately reflected in PM statistical results.

Combined, the above results confirm that the PM approach is generally applicable to genetically-, biochemically- and environmentally induced metabolic responses and that the supplementary metabolomics data tables provided with even relatively old studies contain sufficient statistical information for detecting functional links between such responses.

## Discussion

### Biological pathway mutants are useful for benchmarking phenotype matching tools

In this study, we used a set of *A. thaliana* mutants affected in various steps of the photorespiration metabolic pathway as a tool to test the performance of different similarity scoring methods. Genetic mutants are particularly suitable for this purpose because the nature of their perturbation may be determined or targeted very precisely and they are readily available to the community. This contrasts with other perturbations such as pharmacological enzyme inhibitors, for example, that can have unknown off-target effects (undesired or unknown inhibitions of non-target enzymes) even when they nominally hit the same target.

Theoretically, two mutants with loss-of-function mutations in the same gene in the same genetic background should display essentially the same phenotype and any residual differences in phenotype should represent technical noise (including unavoidable biological effects caused by imperfect replication of experimental design and starting biological state of germplasm). Thus, assuming that there are no significant technical errors within the data, the ideal phenotype matching tool should score matches between such functionally equivalent mutants above matches between functionally non-equivalent mutants. This should be facile if the mutant pairs to be discriminated are functionally unrelated. However, when the mutant pairs represent steps in common biological pathway, the task of maintaining correct matches by correctly discriminating between responses to functionally related perturbations becomes more challenging.

In this study, a demonstration of the challenge of resolving the phenotypes associated with functionally related perturbations was the frequent failure to correctly discriminate between phenotypes of *mtkas* and *shm1* mutants (Table 1). Despite carrying mutations in different genes and being directly affected in different enzymes, *mtkas* and *shm1* mutants have essentially identical metabolic phenotypes because the enzymes they are affected in (GDC and SHMT, respectively) cooperate in the conversion of glycine to serine in reactions that are tightly coupled via a common pool of THFs in the mitochondrial matrix (Prabhu et al., 1996).

Further evidence of pathway connections driving phenotypic similarities that challenge statistical discrimination was seen in the mismatches of *glu1* query mutants to the *dct* reference instead of *glu1* references when *R*^2^ and BWMC were used as similarity measures (Table 1). The GLU1 and DiT2.1 enzymes affected in *glu1* and *dct* mutants are not as directly biochemically coupled as GDC and SHMT. However, DiT2.1 supports GLU1 by its involvement in the import of the 2-oxoglutarate consumed by GLU1 into the chloroplast and the export of the glutamate GLU1 produces (Somerville and Ogren, 1983; Renné et al., 2003). Thus, impairment of either enzyme will cause a similar disruption of photorespiratory nitrogen recycling pathway and lead to similar responses in the metabolome. Effective phenotypic matching tools should be as reliable as possible at discriminating between similar responses and we therefore recommend testing the ability to discriminate biological pathway mutants as a general approach to benchmarking phenotypic similarity scoring methods that aim to detect functional links.

### Quantitative correlation and qualitative overlap: Complementary indicators

Quantitative correlations are often used to assess similarities between molecular response profiles (Langfelder and Horvath, 2012) while Fisher’s Exact Tests have been used widely to calculate the significance of qualitative overlaps between gene sets (Huang et al., 2009) so these were obvious starting points in our hunt for an effective similarity measure. We observed that *R*^2^ and FET2p gave different sets of mismatches in our performance test while the hybrid PM score based on both parameters outperformed either parameter alone (Table 1). Significantly, this suggests that quantitative correlation and qualitative overlap are complementary indicators of functional links between metabolic phenotypes and that statistical similarity measures that utilize both effectively will likely outperform those that utilize only one.

To contribute to the FET2p term of the PM score, metabolites need only be increased or decreased by more than the minimum fold-change threshold (1.5-fold by default) in both the query and reference; there is no requirement for statistical significance and no weighting according to fold-change. The FET2p term can therefore be large in the absence of any strong or statistically significant metabolite responses, as long as the directions of the metabolite responses overlap with the reference. Achieving a high *R*^2^ term does not depend on a query having strong or statistically significant responses either. Taken together, our observations highlight the importance of publishing and sharing complete sets of metabolite responses, including small and statistically insignificant changes, rather than only those responses that have a high fold change or low *p*-value. While individual non-significant trends may seem unworthy of publication in themselves, collectively, they can contain crucial information.

### Facile cross-study comparisons will facilitate the discussion of metabolomics results

When the sequences of new genes or proteins are reported in the literature, it is standard practice to report and discuss some measure of their similarity (e.g., % identity) to already known sequences, as determined using a similarity search like BLAST (Altschul et al., 1990). By contrast, with few exceptions (Fukushima et al., 2014), when new metabolite responses are reported, comparisons to previous results tend to be of a subjective and qualitative nature, comparing only selected features (e.g., “… authors Y also observed an increase in metabolite X…”) and not providing an objective statistical measure of similarity. Metabolomics databases such as MetaboLights (Haug et al., 2013), Metabolomics Workbench, and MetabolomeExpress (Carroll et al., 2010) already contain sufficient volumes of metabolite response data to make PM-like searches a worthwhile and effective means of establishing where newly observed metabolic phenotypes and responses lay in the collective phenomic landscape. What features do new observations have in common with previous ones? Are there any features of a newly observed phenotype which distinguish it from its nearest neighbors in phenotypic space? If a new metabolite response is significantly similar to one or more entries in a public database, this match should be reported because it may point to an underlying mechanistic functional link that may otherwise escape detection. Conversely, if a new response bears no similarity to known responses, this is also worth reporting because it points to an under-explored type of response linked to an under-explored response mechanism just as a gene with no homology to known genes points to an under-explored new class of genes.

### The phenometer is suited to large-scale metabolic phenotypic forward screening

The emergence of fast NGS-based bulk segregant analysis methods has dramatically reduced the time and effort involved in mapping causative mutations in mutants isolated by forward genetic screens (Schneeberger et al., 2009; Hartwig et al., 2012). Thus, these techniques increase the attractiveness of forward genetic screening as a functional genomics gene discovery approach. Forward screens have traditionally relied on visual phenotypic signals such as a visible change in appearance or expression of a visible marker signal. However, high-throughput biochemical assays that allow thousands of individuals to be assayed in practical time frames offer a powerful alternative approach to mine large mutant populations for mutants affected in specific metabolic pathways. In our speed performance tests, we showed that, using the PM, a single metabolic phenotype can be searched against 14 reference phenotypes, giving a reliable diagnosis of any of 14 metabolic lesions in less than 2 s. Thus, high-throughput robotic metabolite extractions combined with, for example, high-throughput direct infusion MS (Koulman et al., 2007), automated data processing and PM diagnosis could potentially provide sufficient throughput to be used for metabolomics-based forward screening.

### Challenges and limitations of the phenometer approach

While our results demonstrate that the PM approach performs well at its task of retrieving from the database metabolite response patterns that are most similar to a query pattern, there are a number of important limitations to this approach that the user should be aware of. Firstly, there is the issue of bias. The simplest bias is that related to database content. The range of potential hits to any query is obviously limited to what is represented in the reference database. Thus, the best hit returned to a query does not necessarily represent the best hit that would be returned if the database contained all data available in the literature. Fortunately, however, the computation of the PM score using familiar statistical concepts and the incorporation of statistical significance testing into PM results means that the significance of returned hits can be interpreted at “face value”. That is, a “strong” hit is likely to reflect a real functional link regardless of whether another response might exist that would give a stronger hit. This is comparable to a strong BLAST match between a query and reference gene which suggests an evolutionary link between them even though closer homologs exist for each gene.

Another source of bias is linked to metabolome coverage (the sets of metabolites represented in metabolic phenotypes). The maximum score achievable for any given phenotype comparison is dependent upon the number of metabolites involved in the comparison. Matches based on more metabolites have more statistical power in the Fisher’s Exact Test and can thus achieve lower FET2p *p*-values and thus higher (–log(FET2p)) terms than matches based on fewer metabolites. While this means that hits are, appropriately, ranked according to the strength of the statistical evidence for their similarity, it does introduce the potential for “technology platform bias” whereby reference phenotypes that have an inherently higher degree of overlap in metabolome coverage with the query have the capacity to achieve a higher score than other phenotypes that have less coverage. Higher metabolome coverage overlap can be achieved, e.g., because both reference and queried metabolome were acquired on the same or similar analytical platform detecting a similar set of metabolites. Fortunately, metabolite response directions and correlations between metabolic phenotypes are not influenced by technology platform. That is, a strong hit will not arise simply because it was acquired on a similar platform to the query. Moreover, the permutation-based significance test (Figures 3 and 4) inherently adjusts for “platform bias” in its determination of statistical significance because it determines the null score distribution by repeatedly shuffling the same set of metabolites as included in the query. Thus, the null score distribution from which *p*_non-bio_ is estimated will reflect the influence of any platform bias arising from metabolite coverage.

Another factor challenging the PM (also encountered with any tool attempting to detecting similarities between independently observed molecular response patterns for the purposes of inferring functional links) is experimental reproducibility. The variable success of our attempts at cross-study matching (Table 4) highlights the fact that similar if not identical perturbations do not always result in significantly similar metabolite responses being reported, even when the species, genetic background, analytical technology, and growth conditions are the same or similar. For example, the failure of the phenotypes reported for *hpr1* by Timm et al. (2012) to match strongly our *hpr1* reference phenotype highlights the low reproducibility of the metabolic phenotype in this mutant. This perhaps reflected a rather high sensitivity to differences in growth conditions or the subtlety of its phenotype (i.e., characterized by weak metabolic changes) causing its phenotypic signal to be obscured by analytical noise.

Taken together, the above observations underline the value in having the highest possible metabolome coverage, standardizing the annotation of unidentified metabolites (sharing of standardized spectral reference libraries) while employing effective biological replication and experimental design to minimize random experimental noise and systematic errors.

## Conclusion

Metabolomics databases are growing rapidly in size and maturing in their development toward becoming analytical tools. By allowing individual datasets to be analyzed and visualized using integrated tools, added-value metabolomics databases like Metabolomics Workbench^3^ are making data more accessible, transparent, and useful. However, to our knowledge, there are currently no other metabolomics databases besides MetabolomeExpress equipped tools allowing data from *independent* studies to be compared, aligned, and thereby mined collectively for interesting patterns. The incorporation of tools allowing cross-study analysis into metabolomics databases offers to make metabolomics databases more than the sum of their parts since the number of comparisons possible between *m* metabolite response patterns is *m*^2^. We hope others find our observations and approaches useful in the development of similar tools.

## Materials and Methods

### Plant genotypes

The line used as the wild-type reference for metabolomics in this study was *A. thaliana* ecotype Col-0. Analyzed mutant lines included: GDC-impaired *mtkas-1* (Somerville and Ogren, 1982; Ewald et al., 2007), SHMT-impaired *shm1-1* (Somerville and Ogren, 1981; Voll et al., 2006), Fd-GOGAT-impaired *gls1-103 and gls1-113* (Somerville and Ogren, 1980a; Coschigano et al., 1998), DiT2-impaired *dct* (Somerville and Ogren, 1983; Renné et al., 2003), rubisco activase-impaired *rca* (Somerville et al., 1982; Orozco et al., 1993), GLYK-impaired *glyk* (Boldt et al., 2005), SGAT-impaired *agt1-1* and *agt1-2* (Somerville and Ogren, 1980b; Liepman and Olsen, 2001), HPR1-impaired *hpr1-1* (Timm et al., 2008), peroxisomal catalase 2 (CAT2)-impaired *cat2-2* (Queval et al., 2007), peroxisomal malate dehydrogenase 1 (pMDH1)-impaired *pmdh1*, and peroxisomal malate dehydrogenase 2 (pMDH2)-impaired *pmdh2*, pMDH1- and pMDH2-impaired double mutant *pmdh1pmdh2* (Pracharoenwattana et al., 2007; Cousins et al., 2008). Seed for these lines were obtained through the Arabidopsis Biological Resource Center with the exception of the *pmdh*, *cat2-2*, and *agt1* mutant lines that were kindly provided by Pracharoenwattana et al. (2007), Graham Noctor (Queval et al., 2007), and (Liepman and Olsen, 2001), respectively.

### Plant growth and tissue harvest for metabolomics experiments

*Arabidopsis thaliana* seeds were allowed to imbibe on wet filter paper and stratified for 5 days. Seeds were germinated and grown on a mixture of potting soil (Debco seed raising mix) and 4 g Osmocote L^−1^ soil (Osmocote Exact Mini; Scotts Australia). Five replicate plants of each genotype were grown under high CO_2_ conditions at 6 mL L^−1^ CO_2_ in a controlled environment growth cabinet at an irradiance of 140 μmol quanta m^−2^ s^−1^ and air temperature of 22°C during the day and 18°C at night, with a day length of 14 h. After 5 weeks, plants were transferred from high (CO_2_) to ambient (CO_2_) (400 μL L^−1^ CO_2_ but otherwise similar) conditions for 2 h before leaves were harvested for metabolomic analysis. Harvesting was carried out by rapidly excising and sealing 50–100 mg of leaf tissue, cut at the base of the petiole, in a 2 ml polypropylene round-bottom safe-lock Eppendorf tube (Eppendorf; Cat. No. 0030 120.094) containing a 5 mm diameter stainless steel ball (Qiagen; Cat. No. 69989) and freezing in liquid nitrogen within 15 s. Harvested samples were kept frozen at −80°C until analysis. The genotypes were analyzed over three separate experiments, each with its own set of wild-type control plants.

### Metabolite extraction

Frozen leaf samples were pulverized in a TissueLyser II bead mill (Qiagen; Cat. No. 85300) for 1 min at 20 Hz. Approximately 30 mg of the resulting tissue powder was transferred and accurately weighed, without thawing, to a new, cold 2 mL round-bottom safe-lock Eppendorf tube (Eppendorf; Cat. No. 0030 120.094). Tubes were kept frozen on a tube rack chilled with liquid nitrogen as five volumes (5 μL per mg fresh weight of tissue) of room temperature extraction medium [85% (v/v) HPLC grade MeOH (Sigma), 15% (v/v) untreated MilliQ H_2_O, 100 ng μL^−1^ ribitol] was added to each tube followed by brief vortexing to give thorough mixing of the solvent and tissue powder. Tubes were placed back on liquid nitrogen until all the samples had been mixed with extraction medium. The tubes were then quickly transferred to an Eppendorf Thermomixer Comfort (Eppendorf; Cat. No. 5355 000.011), rapidly heated to 60°C and shaken at 1400 RPM for 15 min with internal gas pressure being released from tubes by momentarily opening tube lids after 1 min of heating. Tubes were then centrifuged at 20000 g for 10 min to pellet insoluble material and the supernatants transferred to new 2 mL round-bottom safe-lock Eppendorf microfuge tubes (Eppendorf; Cat. No. 5355 000.011). These stock extracts were centrifuged again at 20000 *g* for 10 min to ensure the complete absence of insoluble material and 20 μL aliquots of the supernatants were dried in 2 mL amber crimp-cap autosampler vials (Grace Davison Discovery Sciences; Catalog Number 31811E-1232A) fitted with silanized glass 250 μL low-volume inserts (Grace Davison Discovery Sciences; Cat. No. 983228) using a Labconco CentriVap Acid-Resistant System (Labconco; Cat. No. 7983014) operated at room temperature.

### Metabolite derivatization

Dried metabolite extracts were chemically derivatized by methoximation and trimethylsilylation on a Gerstel MPS2XL Multipurpose Sampler (Gerstel) operating in the PrepAhead mode for automated online derivatization and sample injection. The derivatization procedure consisted of the following steps: (1) addition of 10 μL of 20 mg ml^−1^ methoxyamine hydrochloride (Supelco, Cat. # 33045-U) in anhydrous derivatization-grade pyridine (Sigma-Aldrich, Cat. # 270970) and incubation at 37°C for 90 min with agitation at 750 RPM; (2) addition of 15 μL of derivatization grade *N*-methyl-*N*-(trimethylsilyl)trifluoroacetamide (MSTFA; Sigma-Aldrich; Cat. No. 394866) and incubation at 37°C for 30 min with agitation at 750 RPM; 3) addition of 5 μL of alkane mix [0.029% (v/v) n-dodecane, 0.029% (v/v) n-pentadecane, 0.029% (w/v) n-non-adecane, 0.029% (w/v) n-docosane, 0.029% (w/v) n-octacosane, 0.029% (w/v) n-dotriacontane, and 0.029% (w/v) n-hexatriacontane dissolved in anhydrous pyridine] and incubation for 1 min at 37°C with agitation at 750 RPM. Samples were injected into the GC/MS instrument immediately after derivatization.

### Gas chromatography/mass spectrometry metabolomic analysis

Derivatized metabolite samples were analyzed on an Agilent 5975C GC/MSD system comprised of an Agilent GC 7890N gas chromatograph (Agilent Technologies, Palo Alto, CA, USA) and 5975C Inert MSD quadrupole MS detector (Agilent Technologies, Palo Alto, CA, USA). The GC was fitted with a 0.25 mm ID, 0.25 μm film thickness, 30 m Varian FactorFour VF-5 ms capillary column with 10 m integrated guard column (Varian, Inc., Palo Alto, CA, USA; Product No. CP9013). Samples were injected into the split/splitless injector operating in splitless mode with an injection volume of 1 μL, an initial septum purge flow of 3 mL min^−1^ increasing to 20 mL min^−1^ after 1 min and a constant inlet temperature of 230°C. Helium carrier gas flow rate was held constant at 1 mL min^−1^. The GC column oven was held at the initial temperature of 70°C for 1 min before being increased to 325°C at 15°C min^−1^ before being held at 325°C for 3 min. Total run time was 21 min. Transfer line temperature was 250°C. MS source temperature was 250°C. Quadrupole temperature was 150°C. Electron Impact ionization energy was 70 eV and the MS detector was operated in full scan mode in the range of 40–600 *m/z* with a scan rate of 3.6 Hz. The MSD was pre-tuned against perfluorotributylamine (PFTBA) mass calibrant using the “atune.u” autotune method provided with Agilent GC/MSD Productivity ChemStation Software (Revision E.02.01.1177; Agilent Technologies, Palo Alto, CA, USA; Product No. G1701EA).

### Metabolomics data processing and statistical analysis

All GC/MS data were processed using the online *MetabolomeExpress* data processing pipeline^4^ (Carroll et al., 2010). Raw GC/MS files were exported to NetCDF format using Agilent MSD ChemStation software (Revision E.02.01.1177; Agilent Technologies, Palo Alto, CA, USA; Product No. G1701EA) and NetCDF files were uploaded to the ANU_Badger *MetabolomeExpress* data repository. Peak detection settings were: Slope threshold = 200; Min Peak Area = 1000; Min. Peak Height = 500; Min. Peak Purity Factor = 2; Min. Peak Width (Scans) = 5; Extract Peaks = on. Peaks were identified by MSRI library matching which used retention index and mass-spectral similarity as identification criteria. MSRI library matching parameters were as follows: RI Window = ± 2 RI Units; MST Centroid Distance = ± 1 RI Unit; Min. Peak Area (for peak import): 5000; MS Qualifier Ion Ratio Error Tolerance = 30%; Min. Number of Correct Ratio Qualifier Ions = 2; Max. Average MS Ratio Error = 70%; Remove qualifier ion not time-correlated with quantifier ion = OFF; Primary MSRI Library = “Carroll_2014_Arabidopsis_Photorespiration_Mutants.MSRI”; Add Unidentified Peaks to Custom MSRI Library = OFF; Use RI calibration file specified in metadata file = ON; Carry out per-sample fine RI calibration using internal RI standards = OFF. The Carroll_2014_Arabidopsis_Photorespiration_Mutants.MSRI primary library contains entries derived manually from analyses of authentic metabolite standards run under the same GC/MS conditions as the biological samples as well as entries for unidentified peaks that were automatically generated by MetabolomeExpress while processing the data from the reference photorespiration mutants.

Library matching results were then used to construct a metabolite x sample data matrix with peak areas being normalized to internal standard (i.e., ribitol). As a quality control filter, samples were checked for the presence of a strong ribitol peak with a peak area of at least 1 × 10^5^ and a deviation from the median internal standard peak area (for that GC/MS batch sequence) of less than 70% of the median value. Statistical normalization to tissue mass was not required because chemical normalization to tissue mass had already been carried out by adjusting extraction solvent volume proportionally to tissue mass. For determination of metabolic phenotypes, the mutant/genotype SIR of each metabolite was calculated by dividing the mean (normalized) signal intensity of each metabolite in each set of mutant plants by its mean (normalized) signal intensity in its associated set of wild-type control plants. Statistical significances were calculated by two-tailed Welch’s *t*-tests (*n* = 5) in the *MetabolomeExpress* Comparative Statistics tool. The full dataset has been uploaded to the *MetabolomeExpress* Phenotype Database (MetabolomeExpress Dataset IDs 36, 42, and 43) and will be made publicly accessible upon publication of this article.

### Next-generation genome sequencing

Genomic DNA was extracted using the Qiagen DNeasy Plant Mini Kit following the manufacturer’s instruction. Quality was checked using spectrophotometer and agarose gel electrophoresis. DNA concentrations were determined by the Qubit (Invitrogen) system. Genomic libraries were constructed using the TruSeq^™^ DNA Sample Preparation kit (Illumina) following the manufacturer’s Low-Throughput Protocol. Briefly, 1 μg of genomic DNA was fragmented by Covaris shearing to produce 300–400 bp fragments. After repairing the ends of the fragments to produce blunt ends, 3′ adenylation was performed, followed by ligating distinct DNA adapter indexes to distinct genotypes. The ligation products were enriched by 10 cycles of PCR. The size of the products was analyzed using the Bioanalyzer 2100 (Agilent Technology). The DNA libraries were diluted and pooled so that equal amount of DNA from each genotype was sequenced on a lane of a flow cell, with seven libraries on a single lane. DNA was sequenced using a HiSeq 2000 (Illumina) 100 bp paired-ends reads at the Biomolecular Research Facility at the Australian National University John Curtin School of Medical Research (JCSMR). Reads that had been de-multiplexed and filtered using the instrument manufacturer’s software were supplied by the facility. Alignment of reads from each mutant to the Col-0 reference genome assembly (ftp://ftp.jgi-psf.org/pub/compgen/phytozome/v9.0/Athaliana/annotation/Athaliana_167_protein.fa.gz) available at Phytozome (Goodstein et al., 2012) was performed using BWA (Li and Durbin, 2009). Single nucleotide polymorphisms (SNPs) were detected using SAMtools (Li et al., 2009). The scripts used to align reads and detect SNPs are provided in Data Sheet S1 in Supplementary Material. SNPs within or close to known photorespiratory genes were retrieved from VCF files generated by SAMtools using a custom PHP script to retrieve any SNPs lying between 1000bp upstream of the start coordinates and the end coordinates of the known photorespiratory genes provided in Table S2 in Supplementary Material. The effects of SNPs on protein sequences were predicted using the ENSEMBL Plant Variant Effect Predictor.^5^ The conservation of the affected amino acids across plants was assessed using PipeAlign (Plewniak et al., 2003).

### Protein extraction, electrophoresis and immunodetection

Leaf total protein was extracted with buffer containing 50 mM EPPS, 1 mM EDTA pH 7.8, 5 mM MgCl_2_, 1% PVPP, 1% Triton X-100, and 10 mM DTT. Protein (10 μg) was separated using 4 -12% NuPAGE^®^ Bis-Tris Precast Gel (Inivitrogen), then transferred onto PVDF membrane. The Fd-GOGAT protein was detected with rabbit anti-Fd-GOGAT antibody (Agrisera). An AP-conjugated Goat anti-rabbit secondary antibody (Sigma) and AP-conjugate substrate kit (BioRad) were used for detection of the primary antibody.

## Conflict of Interest Statement

The authors declare that the research was conducted in the absence of any commercial or financial relationships that could be construed as a potential conflict of interest.

## Supplementary Material

The Supplementary Material for this article can be found online at http://journal.frontiersin.org/article/10.3389/fbioe.2015.00106

Table S1 **Genetic lesions in known photorespiration genes in newly isolated A. thaliana photorespiration mutants**.

Table S2 **Table of coordinates for known photorespiratory genes used to detect SNPs in known photorespiratory genes in sequenced mutants**.

Data Sheet S1 **Scripts used to align reads and detect SNPs using BWA and SAMtools**.

Figure S1 **Western blot analysis of GLU1 protein abundance in 17-6E4 mutant**.

Figure S2 **Phenotypic similarity network graph of *A. thaliana* temperature stress time courses from data reported by Kaplan et al. (2004)**.

## Footnotes

^1^http://gmd.mpimp-golm.mpg.de/

^2^https://www.metabolome-express.org/phenometer.php

^3^http://www.metabolomicsworkbench.org/

^4^www.metabolome-express.org

^5^http://plants.ensembl.org/tools.html
