# Supplementary material for: PhenoMeter: A Metabolome Database Search Tool Using Statistical Similarity Matching of Metabolic Phenotypes for High-Confidence Detection of Functional Links
Source: Front Bioeng Biotechnol. 2015 Jul 29;3:106. doi: 10.3389/fbioe.2015.00106 (PMC4518198; doi:10.3389/fbioe.2015.00106)
Supplement: Table S1 — Genetic lesions in known photorespiration genes in newly isolated A. thaliana photorespiration mutants. [file table_1.pdf]

Query mutant ID	Top PhenoMeter hit	Mutated gene	Base change	Phred quality score	Protein change	Correct match?[a]
17-10F4	*gls1-113*	*GLU1*	Chr5: 1136206 G>A	83.1	G1252E	Y
24-2E5	*gls1-113*	*GLU1*	Chr5: 1132955 G>A	148	E579K	Y
32-26C1	*gls1-113*	*GLU1*	Chr5: 1136629 C>T	222	R1306*	Y
24-27C2	*gls1-103*	*GLU1*	Chr5: 1130418 G>A	161	G104R	Y
24-29A8	*gls1-113*	*GLU1*	Chr5: 1131586 C>T	119	P406L	Y
18-29A6	*mtkas-1*	*SHM1*	Chr4: 17833903 C>T	109	E122K	Y
18-21E7	*mtkas-1*	*SHM1*	Chr4: 17833903 C>T	158	E122K	Y
25-35D8	*shm1*	*SHM1*	Chr4: 17833884 C>T	222	R128H	Y
24-14G7	*mtkas-1*	*MTKAS*	Chr2: 1583234 C>T	179	G200R	Y
32-34C7	*agt1-2*	*AGT1*	Chr2: 5540814 C>T	185	R30Q	Y
30-2A7	*agt1-2*	*AGT1*	Chr2: 5539530 C>T	222	G365R	Y
17-59G4	*agt1-1*	*AGT1*	Chr2: 5540273 G>A	145	L183F	Y
17-6E4	*gls1-103*	No consequential mutations in known PR genes				Y[b]

Supplementary Table 1. Genome re-sequencing revealed that top PhenoMeter hits were highly predictive of genetic lesions in query mutants. [a] "Y" indicates that the top *PhenoMeter* hit was to a reference mutant genetically affected in the same enzyme activity (*shm1* and *mtkas-1* both affected in concerted GDC/SHMT activity). [b] The phenotype of 17-6E4 was confirmed to be linked to severe depletion of GLU1 protein. *GLU1* = At5g04140; *SHM1* = At4g37930; *MTKAS* = At2g04540; *AGT1* = At2g13360
