# Supplementary material for: PhenoMeter: A Metabolome Database Search Tool Using Statistical Similarity Matching of Metabolic Phenotypes for High-Confidence Detection of Functional Links
Source: Front Bioeng Biotechnol. 2015 Jul 29;3:106. doi: 10.3389/fbioe.2015.00106 (PMC4518198; doi:10.3389/fbioe.2015.00106)
Supplement: Data Sheet S1 — Scripts used to align reads and detect SNPs using BWA and SAMtools. [file data_sheet_1.pdf]

Supplementary Methods S1:

Example of shell script used to align reads and detect SNPs from genome resequencing of a representative mutant (17-6E4):

```
bwa aln Athaliana_167.fa At17_6E4_GTCCGC_L005_R1_001.fastq >
aln_sa1.sai

bwa aln Athaliana_167.fa At17_6E4_GTCCGC_L005_R2_001.fastq >
aln_sa2.sai

bwa sampe Athaliana_167.fa aln_sa1.sai aln_sa2.sai
At17_6E4_GTCCGC_L005_R1_001.fastq At17_6E4_GTCCGC_L005_R2_001.fastq
> aln.sam

samtools view -bS -o aln.bam aln.sam

samtools sort aln.bam aln.sorted


samtools mpileup -C50 -uf Athaliana_167.fa aln.sorted.bam | bcftools
view -bvcg - > var.raw.bcf

bcftools index var.raw.bcf

bcftools view var.raw.bcf | vcfutils.pl varFilter -D100 >
var.flt.vcf
```
