# Supplementary material for: PhenoMeter: A Metabolome Database Search Tool Using Statistical Similarity Matching of Metabolic Phenotypes for High-Confidence Detection of Functional Links
Source: Front Bioeng Biotechnol. 2015 Jul 29;3:106. doi: 10.3389/fbioe.2015.00106 (PMC4518198; doi:10.3389/fbioe.2015.00106)
Supplement: Figure S1 — Western blot analysis of GLU1 protein abundance in 17-6E4 mutant. [file image_1.pdf]

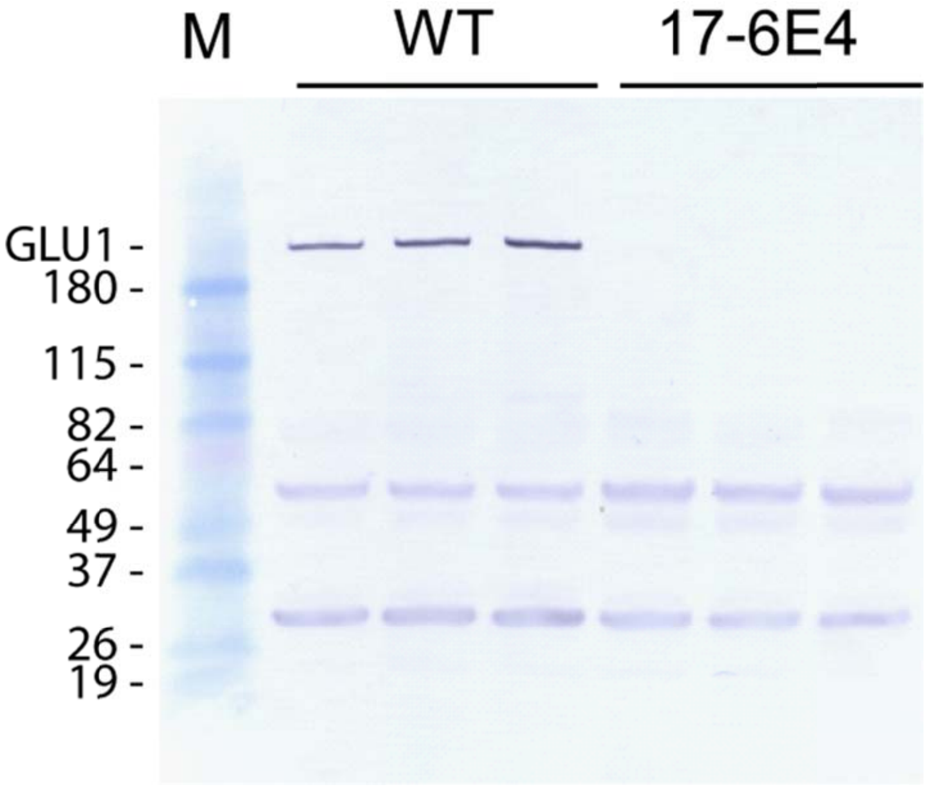

Supplementary Figure S1. Western blotting confirms the *glu1*-like phenotype of *17-6E4* was due to reduced abundance of GLU1 protein. Total protein was extracted from the leaves of three replicate plants each of Col-0 wild-type (WT) and the *17-6E4* mutant line. The proteins were separated by SDS-PAGE, transferred to a PVDF membrane and subject to immunodetection using an anti-AtGLU1 antibody (see Materials and Methods). The blot clearly shows that GLU1, which was readily detectable in the three WT plants, was below the detection limit in the *17-6E4* plants.
