# Supplementary material for: PhenoMeter: A Metabolome Database Search Tool Using Statistical Similarity Matching of Metabolic Phenotypes for High-Confidence Detection of Functional Links
Source: Front Bioeng Biotechnol. 2015 Jul 29;3:106. doi: 10.3389/fbioe.2015.00106 (PMC4518198; doi:10.3389/fbioe.2015.00106)
Supplement: Figure S2 — Phenotypic similarity network graph of A. thaliana temperature stress time courses from data reported by Kaplan et al. (2004). [file image_2.pdf]

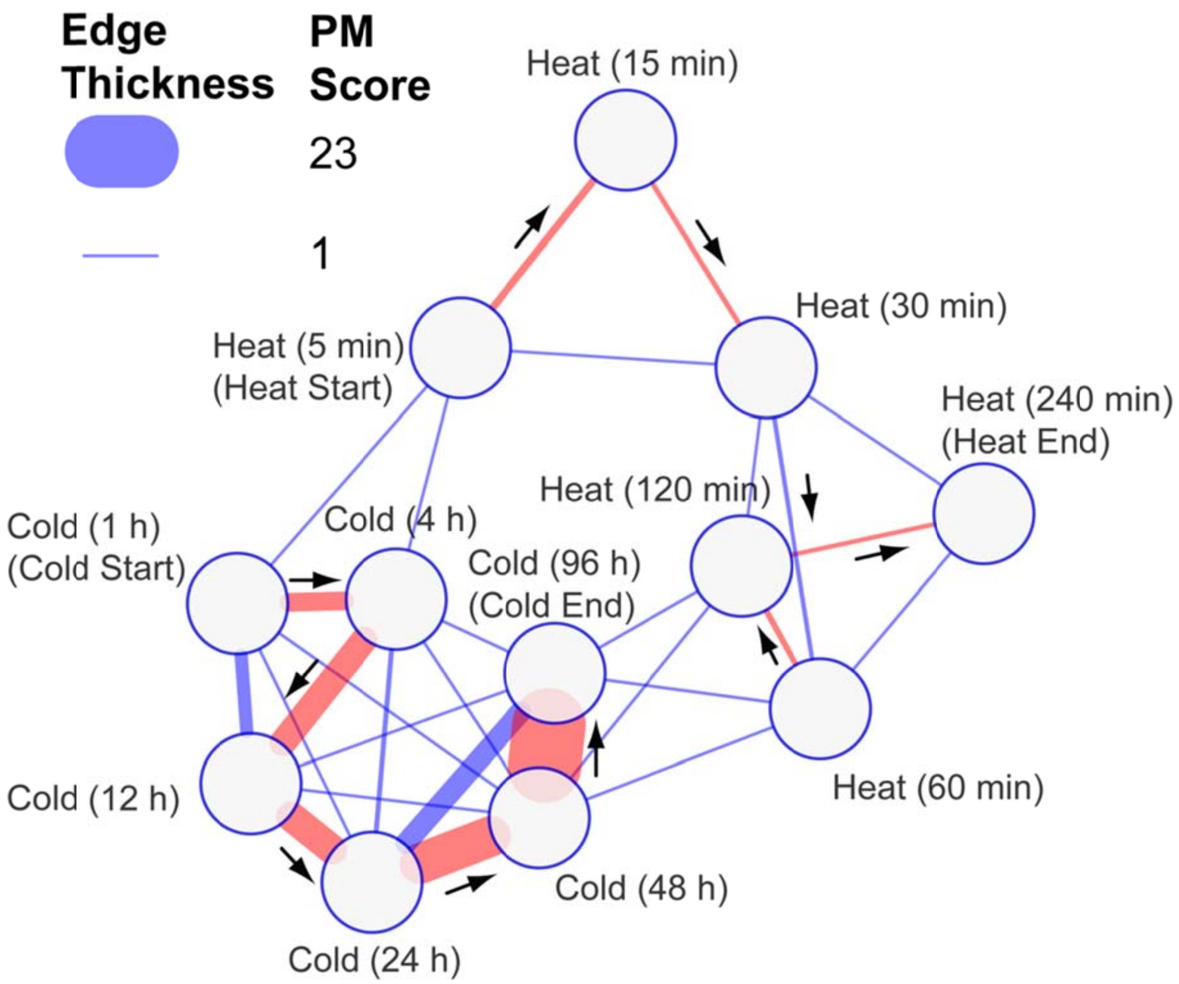

Supplementary Figure S2. Similarity network of Arabidopsis temperature stress metabolic responses. Metabolic responses of *A. thaliana* plants to different durations of exposure to 4 °C cold stress (1, 4, 12, 24, 48 and 96 h) and 40 °C heat stress (5, 15, 30, 60, 120 and 240 min), reported previously by Kaplan et al. (Kaplan et al., 2004) were selected as both bait and prey in a PhenoMeter query to generate a phenotypic similarity network graph with a force directed layout. Edges between nodes represent PhenoMeter matches with FET2p < 0.05 with edge thickness proportional to PM Score. Edges shown in red represent the top match of at least one of the connected nodes. Black arrows highlight the fact that following the pathway of highest PM Scores from the earliest time point (Start) of each stress treatment leads to the latest time point (End) of the same treatment via a route that passes through each intermediate time point in the correct temporal order.
